# Are the stars aligned? Healthcare students’ conditions for negotiating tasks and competencies during interprofessional clinical placement

**DOI:** 10.1186/s12909-023-04636-z

**Published:** 2023-09-08

**Authors:** Tove Törnqvist, Annika Lindh Falk, Catrine Buck Jensen, Anita Iversen, Pia Tingström

**Affiliations:** 1 Department of Health, Medicine and Caring Sciences, Linköping University, Linköping, Sweden; 2 Centre for Faculty Development, Faculty of Health Sciences, UiT The Arctic University of Norway, Tromso, Norway

**Keywords:** Interprofessional education, Clinical placement, Communities of practice, Focused ethnography

## Abstract

**Background:**

Healthcare students must learn to collaborate across professional boundaries so they can make use of each other’s knowledge and competencies in a way that benefits the patient. One aspect of interprofessional collaboration implies negotiating what needs to be done and by whom. Research, focused on the conditions under which students perform this negotiation when they are working together during interprofessional clinical placement, needs to be further developed. The study therefore aimed to explore students’ negotiation of tasks and competencies when students are working together as an interprofessional team during clinical placement.

**Methods:**

The study was designed as a focused ethnographic observational study. Two Nordic sites where final-year healthcare students perform clinical interprofessional education were included. Data consists of fieldnotes, together with informal conversations, group, and focus group interviews. In total, 160 h of participating observations and 3 h of interviews are included in the study. The analysis was informed by the theory on communities of practice.

**Results:**

Students relate to intersecting communities of practice when they negotiate what they should do to help a patient and who should do it. When the different communities of practice align, they support students in coming to an agreement. However, these communities of practice sometimes pulled the students in different directions, and negotiations were sometimes interrupted or stranded. On those occasions, observations show how the interprofessional learning practice conflicted with either clinical practice or one of the student’s profession-specific practices. Conditions that had an impact on whether or not communities of practice aligned when students negotiated these situations proved to be ‘having time to negotiate or not’, as well as ‘feeling safe or not’.

**Conclusions:**

Final-year healthcare students can negotiate who in the team has the competence suited for a specific task. However, they must adapt their negotiations to different communities of practice being enacted at the same time. Educators need to be attentive to this and make an effort to ensure that students benefit from these intersecting communities of practice, both when they align and when they are in conflict.

## Background

Much of the work in health care requires collaboration, both within one’s own area of expertise and between different areas [1–3]. Good collaborative skills are required to ensure patient safety and effective work. This applies to interprofessional collaboration (IPC) in particular, as it involves working together across professional boundaries towards the patient/client’s goals [3]. Collaborating across professional boundaries implies partially sharing areas of knowledge with colleagues of different professions while being an expert in others. This places special demands on good collaborative skills, such as understanding the relationship between one’s own area of knowledge and others’ areas of knowledge [4, 5].

At the same time, there are multiple intersections where several professions and/or persons have knowledge suited for a specific task and may thus be considered for it. This also means that there are situations that call for negotiations as to on which profession, or rather practitioner, will benefit the patient the most in each situation. To be able to negotiate who should do what goes back to the call for collaborative skills. According to the IPEC Core competency framework [4] a healthcare professional should be able to, for example, “recognize one’s limitations in skills, knowledge, and abilities.” They should also be able to “communicate with team members to clarify each member’s responsibility in executing components of a treatment plan or public health intervention.”

To meet these demands of collaborative skills and be able to negotiate competencies, education is required [2, 6, 7] and is regulated in public laws [8, 9]. Even at the undergraduate level, students need to be given the opportunity to “*learn with, from, and about each other to enable effective collaboration and improve health outcomes.“* [3]. Interprofessional education (IPE) serves students with opportunities to learn together across study programs. IPE is based on the idea that students need to practice collaboration with other students who share the same goal while having a different focus based on their specific expertise. For that reason, it has been argued that learning theories derived from the socio-cultural and constructivist perspectives are suited to serve as theoretical foundation for IPE. Situated learning, and more specifically communities of practice (CoP) which builds on situated learning, are examples that has been suggested as favourable in the effort to support IPE theoretically [10, 11]. CoP has been applied within this study and is focused on how people organise themselves into groups based on shared interests and ways of doing things. Members of a CoP learn by sharing and investing in a domain of interest, they join activities and establish relationships, and they develop a shared repertoire of experiences and ways of doing things. As there are multiple CoPs focused on subjects that are of relevance to a healthcare student, the members must also relate to these other CoPs. There are boundaries between the CoPs, which implies that members must negotiate and refine their knowledge when encountering other CoPs. Wenger [12] emphasises that the boundaries between different CoPs hold great potential for learning. The lessons lie in the negotiation between and across boundaries and can be supported by members who are engaged in multiple CoPs. By transferring, connecting, and explaining different elements of other CoPs, these members are brokering the negotiation and are therefore referred to as ‘brokers’.

Research on IPE has so far informed us that students report gaining a better understanding of interprofessional collaboration after doing IPE [13]. They also report gaining a better understanding of different roles and responsibilities [14, 15]. This helps us understand students’ perspectives and experiences but does not tell us as much regarding how students enact negotiations during interprofessional clinical placement. We must therefore continue to deepen our collective understanding of how students come to terms with who has the competence that will benefit the patient the most. This gap in knowledge also includes what conditions students are under when they are negotiating tasks and competencies. It calls for observational research focused on exploring situations where students discuss their different competencies and decide who will be responsible for a specific task. The aim of this paper is therefore to explore students’ negotiation of tasks and competencies when working together as an interprofessional team during clinical placement.

## Methods and study design

The study is carried out in collaboration with researchers from two Nordic universities, and uses a design centred on focused ethnographic approach [16, 17]. Data has been collected through field observations at two different sites: a health centre (HC) in Norway, and an interprofessional training ward (IPTW) in Sweden. Both sites serve students with opportunities to learn interprofessional collaboration.

### Research setting

The study includes two different learning sites: the HC and the IPTW. See Table 1 for an overview of the two sites. Changes to the set-up of the IPE activity at the HC were made between the first and second/third data collection periods. Therefore, in Tables 1, 2 and 3, the first observation period at the HC is described separately from the second and third periods.

**Table 1:** Overview of the research setting

	HC1, Norway	HC 2 + 3, Norway	IPTW 1 + 2, Sweden
Type of health care facility	Community-based intermediate health care.		Hospital-based orthopaedic ward.
Duration of IPE	2 days	2–4 days	2 weeks
Tasks	Intermediate care of 1 geriatric patient, including having a dialogue with and examining the patient, morning routines, follow-ups, handing over the patient to other colleagues, writing a report.	Intermediate care of 2–3 elderly patients with complex health and care needs, including having a dialogue with and examining the patient, morning routines, follow-ups, handing over the patient to other colleagues, writing a report.	Specialist care of 4–6 admitted patients with a variety of orthopaedic issues, including morning and evening routines, medical assessments, rehabilitation, admission, discharge, and more.
Work hours	Day 1: 10.00–15.30 Day 2: 08.00–14.15	Day shifts: 08.00–15.00	Day shifts: 6.45–15.30 Evening shifts: 14.00–21.00
Supervisors	Team of supervisors coming and going.	Team of supervisors coming and going.	Team supervisors present throughout the shifts. Uniprofessional supervisors available daily.
Examples of learning outcomes (freely translated from the original Norwegian/ Swedish learning outcomes)	After graduation, the student can: - Apply their own subject knowledge in collaboration with students from other professions, patients, and other partners. - Initiate, plan, implement, coordinate, and evaluate their own professional work in collaboration with other students and contribute to joint decisions in interprofessional work. - Demonstrate the ability for interprofessional cooperation based on values such as respect for others, patient centeredness, equality, and recognition of each other’s competence.		On completion of the IPTW, the student should demonstrate: - Ability to contribute to the team’s planning, implementation, and evaluation of good, safe, and effective care. - Knowledge and understanding of one’s own and other professions’ competencies. - Ability to evaluate the care process based on evidence and guidelines that apply to the organisation. - Ability to actively present and orally reflect on teamwork and interprofessional competence in relation to evidence and good, safe, and effective care.
Facilities being used	A student room outside the ward A patient room inside the ward A nursing station at the ward	A student room outside the ward A patient room inside the ward A nursing station at the ward	A wing of their own at the orthopaedic ward with six patient rooms A dedicated team room A dedicated medical student room A conference room for ward rounds and reading reports

The HC is a newly established municipal intermediate health care facility where patients are treated by interprofessional teams. Patients admitted to the HC need advanced but not specialised hospital care. The IPE activity is based on pragmatic approach as students who are at the HC for program-specific clinical placement devote 2–4 days of their clinical placement to IPE. Supervisors focused on facilitating teamwork are available and at hand. The main change between the first and second/third data collection periods were the number of patients students worked with, from one patient to two or three. During the second data collection period, students also worked together for four days, while in the first and third they worked together for two days.

The IPTW is a well-established form of clinical IPE first introduced in the mid-1990s [18]. Students from different health care programs work together at a hospital ward where a wing of the ward is specially dedicated to them. They have full responsibility for the admitted patients during day and evening shift, between which the two teams alternate. During nights and weekends, regular staff are responsible for the patients. Supervisors are present and/or available at hand throughout the shifts and are focused on facilitating both teamwork and uniprofessional work. No changes between the set-up was made between the different observation periods.

### Participants

Students from 4 to 6 different healthcare programs participated in the study and were divided equally into 2–3 teams at each site, see Table 2. Ahead of data collection, an e-mail was sent to students joining the different IPE activities with information about the study. It also informed the students on the principle of consent [19], that they would sign a consent form if agreeing to participate and that they could withdraw their consent at any time during the observations without having to explain why or risk any consequences. At the start of the respective IPE activity, the same information was recounted orally, and the participants signed an informed consent form.

**Table 2:** Participating students

	HC1, Norway		HC 2 + 3, Norway		IPTW 1 + 2, Sweden	
	*N*	*Semester (out of)*	*n*	*Semester (out of)*	*n*	*Semester (out of)*
Dentistry	2	9(10)	-	-	-	-
Medicine	3	11(12)	5	11/12(12)	5	9(11)
Nursing	3	5(6)	17	5/6(6)	10	6(6)
Occupational therapy	1	5(6)	1	5(6)	2	6(6)
Pharmacy	2	9(10)	6	7/8(10)	-	-
Physiotherapy	-	-	3	5/6(6)	3	6(6)

Students at the HC had one prior experience of IPE. They had started their education with a 10-credit course including themes like IPE, the healthcare system, ethics, communication, academic writing, and scientific methods. Students at the IPTW had multiple experiences in IPE ahead of the IPTW, including a 6-credit IPE activity during their first semester, and a 3 credit IPE activity in the middle of their study programs. Both IPE activities were theoretical studies and include themes like ethics, health, and improvement science.

### Data collection

Data collection includes participatory fieldwork and different forms of interviews. Fieldwork forms the basis of ethnographic research. Through observations, the researcher can generate a deep understanding of multiple aspects of the context they investigate, not just people’s statements about their experiences [20]. A focused approach means that the observations are carefully selected in light of previous knowledge. Data collection periods are usually relatively short, and chosen pragmatically based on knowledge gaps and/or theoretical assumptions [17]. Focused ethnography is highlighted as particularly suitable for observational studies in medical education [16].

At the HC, data was collected during three periods; the first by authors TT and ALF, the second and third by CBJ. During fieldwork, TT and ALF observed one team each from morning-to-afternoon during the two days of IPE. Author CBJ alternated between the teams during all morning-to-afternoon shifts throughout the IPE-activity. At the IPTW, data was collected during two periods by TT, alternating between the teams for six and nine days respectively. Observations during both morning and evening shifts were undertaken. See an overview of the data collection in Table 3.

**Table 3:** Overview of data collection

	HC1, Norway	HC 2 + 3, Norway	IPTW 1 + 2, Sweden
Data	Fieldnotes Drawings Group interviews	Fieldnotes Drawings Focus group interviews	Fieldnotes Drawings Informal conversations
No. of observed teams	2	5	4
Students per team	5	5–6	5
Observations	Full-time observation during both days.	Selected situations throughout the observation periods.	Selected situations throughout the observation periods.
Observed hours	Approximately 22 h in total	Approximately 42 h in total	Approximately 96 h in total
Interview hours	42 + 35 min	25 + 81 min	-

Situations to observe were chosen based on the aim of the study and what situations were believed to generate rich data. Of particular interest were situations when students had to discuss what to do and who should do it. Observations also followed the daily routines at the respective ward to get a sense of the student’s daily endeavour and thereby identify occasions when students were negotiating with each other. Observations took place at different locations, in allocated team rooms, at the nursing station, in corridors, patient rooms (when applicable), and the patient dining room. The students at the HC mostly worked together as a whole team, enabling us to observe most situations throughout the respective observation period. Only occasionally did the students separate and divide tasks between themselves which meant having to prioritise what situation to observe. At the IPTW, students worked together in smaller groups rather than in a complete team. Consequently, what situations to observe were prioritised on a regular basis. Priorities were made based on what data had been generated and still needed to be generated. Each observation session lasted between 4 and 7 h, including minor breaks taken by the researcher.

Fieldnotes were taken by hand, and when possible, by computer. Verbal memos were occasionally recorded to support later transcription of the fieldnotes. Transcripts were written in close connection to the observations, ensuring that they were more detailed than the initial notes. Drawings of how students, supervisors, and patients were seated and moved around in the rooms were included to support the fieldnotes. Participants are pseudonymised in both fieldnotes and transcripts by being referred to as, for example, ‘NurseStud1’ or ‘OTSupervisor1’.

Interviews and informal conversations were also conducted during data collection. At the IPTW, informal conversations with individual students were held when appropriate. These conversations concerned clarifying the students’ backgrounds, expectations, and how they perceived certain situations. At the end of the first round of data collection at the HC, group interviews with the respective team were conducted. After the second and third data collection periods, one focus group interview per period was conducted at the end of the IPE activity. The groups consisted of 5 and 6 students who were recruited purposefully to ensure representation from the different teams and professions involved. All forms of conversations and interviews were conducted with the purpose of reflecting more closely on situations that had occurred during the respective IPE activity. The ambition was to find out more about how students were thinking about aspects that had been observed. An interview scheme was used as a structure; however, this was used as support to address larger questions and was not followed exactly. Interviews were transcribed verbatim.

The combination of data from different collection periods is chosen to show a variation of how IPE is enacted during clinical placement. The purpose is to contrast and complement different arrangements to obtain a rich and complex dataset.

Data collection was conducted in part during the COVID-19 pandemic. Consequently, restrictions applied at the different sites had to be followed. No interactions between students and patients could be observed at the IPTW, as the patients were isolated in their rooms and no unauthorized persons were allowed there. Also, the first observation period at IPTW had to be interrupted half-way, as there were confirmed cases of infection within the observed group. At the HC, observations were carried out as planned.

### Data analysis

The analysis is based on an iterative, cyclic, and self-reflective process going back and forth between the empirical dataset and writing the paper [17]. The analysis was conducted in collaboration with all authors. Team-based ethnography enables the researchers to share data, involve different perspectives, and cross-check analyses [21, 22]. As this is a Nordic study including data from two different countries, it also enabled verifying language and cultural understandings when being unsure.

**Fig. 1:**
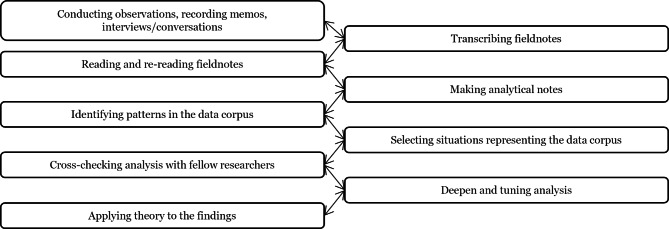
Overview of the analytical process applied. (Modified version of figure by Törnqvist et al. [26])

The analytical process also included applying and discussing theoretical concepts to interpret the empirical findings across the sites. Applying theory to the research process enables a deeper understanding of the empirical data [23, 24] and has been highlighted as an important aspect of interprofessional research [23, 25]. Theoretical concepts derived from CoP was applied during the analysis to aid us in understanding and discussing the empirical findings. See Fig. 1 for an overview of the analytical process applied in the study.

The analysis began during fieldwork when prioritising what situation to observe, and continued to develop throughout the entire process [20]. During fieldwork, the purpose of the overall research project guided preliminary and intuitive interpretations of the situations observed. Part of this analytical approach is the ethnographic ‘hunch’ which Pink [21] describes as moments when the researcher/s realise they have found something significant. The hunch then turns attention to a theme that endures throughout the process. Different hunches—such as students being insecure about their roles— appeared during fieldwork and guided what was paid attention to. During and in-between data collection periods, discussions between the researchers added to this by guiding our attention.

After completing data collection, the analysis shifted to focus on the specific aim of this paper. The analysis was mainly carried out by the first author, with the other authors cross-checking and discussing findings. Discussions between authors were carried out both digitally and in person when visiting each other for extended periods. Inspired by Abrandt Dahlgren and Bjuremark [27], the data was also presented and discussed in seminars with fellow researchers.

The data from observations was read through multiple times to identify situations where at least two students from separate study programs discussed different tasks in relation to differing student competencies. The identification was also inspired by our theoretical standpoint, and the idea that negotiations occur at the boundaries of CoPs. Analytical notes [20] were taken throughout the entire process, and were documented in the form of writing, verbal memos, or mind-maps. The notes included applying theoretical concepts to the findings as a way of abstracting observations from different IPE activities. The concepts were narrowed down to shared repertoires and brokers, as these explain ways of doing things in a CoP and how members of multiple CoPs can connect knowledge from different CoPs.

Parallel to the analysis of observed situations, the interview transcripts were read multiple times. Notes on what students said about different situations and subjects were matched and compared to the fieldnotes. This enabled us to combine different perspectives on the same situation; both what was observed and the students’ own narrative.

The analytical process continued by alternating between revisiting the fieldnotes and interviews, searching for themes and familiar situations across the two sites, discussing, applying theoretical concepts, and then, finally, writing the paper.

## Results

Throughout the different IPE activities, students discussed what should be done and by whom. They did this by contemplating each other’s knowledge competencies and possible interventions. They also contemplated what would be best for the patient. However, the patient’s own perspective was not regularly expressed during the discussions.

Often but not always the students agreed as to what should be done and by whom. It also varied in terms of how long it took them to agree and decide upon a course of action. The findings show that this is a result of a clinical CoP, and an interprofessional learning CoP intersection. Each student also had to relate to a profession-specific CoP of their own. The analysis shows that the intersecting practices shaped conditions for the students’ negotiations. Two conditions stood out; the first is referred to as ‘having time to negotiate or not’, and the second as ‘feeling safe or not’. Both include aspects that enable or hinder students’ negotiations.

The excerpts below serve as examples of situations significant to what was observed across the two sites. Fieldnotes and quotations are translated from the original Swedish or Norwegian.

### Having time to negotiate or not

When the students had time to discuss the situation at their own pace, they negotiated their way to a decision on what to do and who should do it based on their knowledge and competencies. In other cases, the students’ time was limited, and competency-based negotiations were left stranded. However, there were times when the students still had to make a decision, and instead of focusing on competencies decisions were based on who in the group was currently available.

Excerpt 1 exemplifies a situation where time was limited, and students’ competency-based negotiations were hindered. The students are working their first dayshift. They have just had a handover from the night staff and discussed what needed to be done during the day. They begin to divide the patients among themselves. A nursing student initiates a negotiation by requesting help regarding mobilising a patient.

OTStud1 begins and suggests a patient she can start with, framing it as an opportunity to perform an ADL assessment. PhysioStud1 quickly looks up and says, “We can go in together, we didn’t have time for that yesterday”. OTStud1: “Yes”. NurseStud5 tabs in and looks at PhysioStud1 while saying, “I need help with patient X, I’m unsure about mobilisation.“ PhysioStud1 doesn’t respond, looks around the room instead. NurseStud3 says he can help. NurseSupervisor2 cuts in and says, “If you’re going to discuss your patient at the ward round and you don’t see him/her in the morning.“ NurseStud3 suggests in a problem-solving tone: “But I can go in there anyway and say hello.“ NurseSupervisor2 continues: “Or you three [looking towards the nursing students, sweeping her gaze over all three] take your patients and then you three [moving the gaze to the other side where OTStud1, PhysioStud1 and MedStud3 sit] split between the patients. Because you [looking at OTStud1 and PhysioStud1] may not need to go in together.“ The students ”Mm…”.

OTStud1 suggests: “I could go to patient Y instead and do an ADL assessment.“ NurseStud5 looks at OTStud1 and asks: “How long do you need?“, OTStud1: “Not very long.“ NurseStud5: “Mm, because I’m unsure about mobilisation.“ Still no response from the other students.

NurseSupervisor2 looks up at MedStud3 and asks, “Would you be able to go in there?“, MedStud3 nods and “Mm.“ NurseSupervisor2 continues: “And then there are supervisors.“ NurseStud5: “Mm, because I’m unsure about mobilisation in bed after surgery, how to think when helping a patient to get up and such.“, NurseSupervisor2: “[Name of supervisor] can help you.“, NurseStud5: “Mm, good.“ They fall silent. NurseSupervisor2: “Mm, but you have the situation under control.“ The students: ”Yes.” They fall silent and start preparing different things on their own. Shortly after, most of them get up and start exiting the room. On the way out, PhysioStud1 comments: “Oh, it’s always so messy.“

Excerpt 1 IPTW1Team2.

The situation shows how the supervisor cuts in early, and the students’ competency-based negotiation is partly interrupted. The nursing student persists in his request but is interrupted again and they divide the patients based on traditions known from the nursing profession.

In other cases, students had time to discuss what to do and who had the appropriate competence for the task or decision. Together with some support by their supervisors they took their time to figure out who has the competence best suited for the job. Excerpt 2 shows an example where the students start a discussion among themselves, are asked questions by the supervisor, go off to find more information, and then the discussion continues together, resulting in a decision on what to do with a patient’s care needs. It is the second week, and team 1 is working dayshift. They have been busy all morning. MedStud4 and NurseStud7 are sitting in the allocated team room (their version of a nursing station) and are discussing whether to pull a patient’s catheter. The excerpt shows how MedStud4 explicitly acknowledges that nurses are experts on the matter, while the nursing student passes the question back to the medical student. Through the process they discuss and test different arguments.

MedStud4 turns to NurseSupervisor5 who is also sitting in the room and says they would like his help in assessing whether to pull the catheter. NurseStud7 tabs in: “I say no.“ [emphatically]. NurseSupervisor5 asks: “What are you thinking?“ NurseStud7 briefly explains her thoughts. A discussion follows, where both students put forward their arguments for what to do. The focus is on infection risk and difficulty emptying the bladder if the catheter is kept in too long (MedStud4’s argument for pulling it), versus discomfort due to wounds (NurseStud7’s argument for waiting).

NurseSupervisor5 asks: “I’m thinking [NurseStud7], who do you think you could ask? This is a big hospital, who would you turn to if you don’t know yourself?“, NurseStud 7: “The Urologist”, NurseSupervisor5: “Yes, exactly”. There’s a moment of silence before NurseSupervisor5 continues: “We have to make an actual assessment.“ The discussion continues and ends temporarily with NurseStud7 suggesting that MedStud4 should accompany them to the patient, but MedStud4 says “No, I don’t have to look, I trust you two. It’s usually the nurse who does this.“ NurseStud7 and NurseSupervisor5 go off to the room next door for profession-specific supervision.

20 min later, NurseStud7 and NurseSupervisor5 come back, NurseStud7 has called the urologist while they were off. They talk when entering the room, NurseStud7: “So it can stay put until tomorrow?“ NurseSupervisor5: “Yes.“ NurseStud7: “So now it’s up to me to decide?“ NurseSupervisor5 confirms and when he exits NurseStud7 sighs, “Damn it.“

MedStud4 enters the expedition and asks what the urologist said. NurseStud7 recounts the call, then asks: “But what do you think, I think it can stay”. The discussion continues again, MedStud4: “Mm, for what reason?“, NurseStud7 continues with the same arguments as before. MedStud4 asks: “Did they know which patient you were calling for”, NurseStud 7: “Yeah yeah”, MedStud4: “What did they say?“, NurseStud7 recounts more about what they said, then asks “Do you want us to pull it today? We can pull it today.“ MedStud4: “For what reason should we leave it for tomorrow? The wound will not be healed until tomorrow.“

They keep arguing back and forth. The discussion rounds off by MedStud4: “You’re the one making the decision, we can ask [NurseSupervisor5]”, NurseStud7: “He said I have to decide.“ They eventually compromise that they should leave it in place until the afternoon and pull it then. MedStud4 comments: “Great that you called the urologist. Then we’ve learned something today; you can pull a catheter even if there’s a wound.“

Excerpt 2 IPTW2Team1.

### Feeling safe or not

The space being perceived as safe or not was another condition that either enabled or hindered students in negotiating tasks and competencies. In the previous excerpt, apart from the time aspect, it can also be observed how trust was given to each other. Showing trust and confidence in each other proved to be one way of creating a sense of safety. In excerpt 2, the medical student expressed confidence in the nursing student and thereby nudged her to shoulder the role as the expert in this situation. At the same time, a certain uncertainty can be discerned in the nursing student. She seemed to be seeking some form of confirmation from the medical student. In several cases, it was possible to observe similar situations, where the students expressed insecurity and that they were outside of their comfort zone. It was observed how they dodged tasks and that they appreciated when one of the other students took charge. They could also express confusion between their profession-specific tasks and the team’s common tasks. For example, one of the physiotherapist students at the IPTW expressed in an informal conversation that it was difficult to understand the relationship between caring work and physiotherapy.

Excerpt 3 illustrates a situation where one of the dental students expresses discomfort about being with the patient when he/she they get up in the morning. The team had divided the work between them and first distributed what each nursing student would do, then they divided the rest according to which tasks mostly related to their competencies. They are sitting together at a nursing station. They’ve just had a handover from the night shift and are waiting for time to pass before its time to see the patient. While waiting, they loosely talk about what to do during the day.

NurseStud1. looks over at DentalStud1 and says, “So it’s the two of us who start”. NurseStud2 comments “Yes, there’s no point in being more people” before DentalStud1 says “It’s really beyond my competence”. She gives the impression of being insecure, even uncomfortable. One of the nursing students comments “Yes it’s important to see what happens”. It is mostly NurseStud1 talking and pushing the conversation which is a bit low-key. NurseStud2 reminds NurseStud1 to observe how the patient cleans the prosthesis and that they should search the bathroom for prosthetic paste.

They are unsure of what to do while they wait. Someone suggests drinking coffee but does not leave to get it. Instead, they continue to chat about what’s to come. DentalStud1 tells NurseStud1 “I’ll let you steer”, referring to the morning work they are going to do. NurseStud1 notes “You’re not comfortable with it”. DentalStud1 replies that it is not within her professional scope, “Not my job, I am a dental student”.

They proceed to shortly mention an ADL-index they’re supposed to fill in later. NurseStud1 says they can go to the patient soon. MedStud1 asks him to see whether the patient is in pain and if so, does the patient take medication for the pain. While saying it, MedStud1 looks at Pharmacy1 as if to confirm or check they’re in agreement. MedStud1 also asks NurseStud1 to observe if the patient is breathing heavily. MedStud1 continues, notes that they have plenty of time until the next scheduled time with the supervisor.

They’re interrupted when Supervisor1 enters the room. Says she’s available if they need her and that they can go get her when they’re about to start writing in the patient record. She leaves and NurseStud2 asks if it’s time to start. Everyone gets up and walks away.

Excerpt 3 HC1Team1.

The situation at the nursing station is directly followed by the nursing student and dental student going to see the patient. The situation played out with the nursing student taking the lead and doing much of the work, while the dental student stood in the background letting the fellow student do his work. Much of the situation took place in the bathroom with the door closed. The dental student stood outside the door, looking down at the floor or out into the empty space. Whenever the nursing student needed something outside of the bathroom the dental student served him with it. Later in the situation, the other students join. The medical and nursing students do different profession-specific tasks. When done, the medical student leaves and let the others complete their tasks. The dental and pharmacy students stand up against the wall without tasks most of the time.

During the group interview, the dental student referred to the situation and expressed that *”We’re here for interprofessional collaboration, but I felt like it* [morning work] *wasn’t included in my part of the collaboration in a way. Maybe it’s a little bit, maybe it was a little bit harsh to say that and that I expressed that I’m not going to help here* [laughter]. *Eh, yes I thought it worked, eh, when we got in there* [the patient’s room], *you took that role, it was so natural as well, and very nice because. I don’t know what to do, what to do then.”* This exemplifies how the different CoPs are not aligned and create a sense of insecurity.

In a different interview, a physiotherapy student responded to a question on why everyone should engage in their collaborative work. The response also indicates how a sense that you have something to contribute creates a form of safety: *“[I] Believe if everyone feels like they kind of have something to give [Pharmacy2: yes, good point] that it’s easier to kind of, say their opinion and their point of view [Pharmacy2: engage a little yes, agree], yes.“* (PhysioStud1, HC2.)

A different kind of safety was also expressed during one of the focus group interviews at the HC. The students were asked about the competencies in the group and whether they had felt a lack of any specific competency. It led them to reflect upon possible aspects of the patient’s health and care needs that they might have overlooked due to the lack of some competencies.

“But I feel that if we had, for example, a speech therapist, they would have seen their things that they could work on and then we might have—more things would come out and so you kind of take what you have then [PhysioStud1: Yes] Pharmacy2: “Yes, that’s well said.“

– NurseStud8, HC2.

Yes, well it becomes the case with different professions, that you notice things much better when you are familiar with it, what you work with.

– NurseStud2, HC2.

At multiple times, students expressed appreciation for being able to discuss each other’s competencies. There was a situation at the HC were the student team gathered for a review of the medication list of one of the patients. The students were so invested in the discussion they seemed to go into a special mode, leaning over the table to get closer. It started with the medical and pharmacy student initiating the discussion between themselves and ended up with the whole team discussing the situation based on questions derived from each profession’s area of expertise. The pharmacy student was placed in centre of the discussion, supplying answers to different perspectives. Afterwards, in the focus group interview, one of the medical students expressed “*that medication review was so good because, well, you* [Pharmacy2] *were there.”*

## Discussion

The study focuses on students’ negotiation of tasks and competencies when working together as an interprofessional team during clinical placement and is informed by the theoretical lens of communities of practice (CoP). Previous research has shown that IPE involves many elements that need to fit together for the outcome to turn out as intended. If not, the students risk becoming stranded between different ongoing practices [26]. Although not stranded between practices, this paper shows that students’ negotiations are related to the conditions given by intersecting CoPs. Applying CoP and the concepts’ shared repertoire and brokers adds value to the result by abstracting the findings and enabling conclusions from a complex dataset. Despite using two IPE activities situated in different contexts, CoP theory has offered explanations about the students’ negotiations and made it possible to conceptualise our understanding of them.

Clinical placement is a complex form of education. It is the space where the clinic meets education, and multiple processes are going on at the same time [28, 29]. Our findings show that there are several practices intersecting—such as interprofessional and clinical practices—when students negotiate what to do and who should do it. When aligned, the practices enable students not only to negotiate but also to agree on what to do and who should do it. When not aligned, the students’ negotiations are interrupted, and they do not come to terms with whose competence benefits the patient and/or the situation the most. As several practices pulled the students in different directions, we could observe how the students made decisions that did not regard professional competencies, but were focused instead on how someone had to do it or how it was as good as it could be in that situation. In this way, the arrangement of the IPE activity includes conditions that do not match the learning objectives [8, 9], or interprofessional competency frameworks [4]. Interprofessional education is supposed to ensure that students are learning about roles and responsibilities. In the backdrop of our findings, efforts must be made to ensure that the practices are aligned.

At the same time, it should be acknowledged that there is potential for learning when the practices are not aligned. Tensions between different practices serve as good opportunities for learning [12]. If there are tensions between different CoPs, the students must inevitably negotiate and educators can use this consciously to create learning opportunities. In his early work, Wenger acknowledged that a CoP exists in a landscape full of CoPs [12, 30]. When seen from the perspective of professions, this landscape constitutes a professionals body of knowledge. No CoP can capture the full set of knowledge, skills, and competencies that are relevant to a professional. Instead, they exist in this landscape, and the professional can engage actively in some CoPs, while other CoPs remain at the periphery of their knowledge base.

This way of understanding the nature of learning-in-practice applies to this study’s results. The students, who are from multiple study programs, are engaged in different CoPs, some of which are exclusive to their professions. Others are shared between the students, such as the specific clinical ward and the IPE practice. For example, the dental student in excerpt 3 argues that morning work is not included in her normal tasks. This can be understood as a conflict between the profession-specific CoP and the interprofessional CoP. Another example is seen in excerpt 1, where clinical practice calls for the students to interrupt their negotiation, thus forming a conflict between the clinical CoP and the interprofessional CoP. In both situations, there are conditions that hinders the practices from aligning. This is important for educators and researchers to note. There is a risk that it will be difficult for students to engage in the interprofessional CoP unless considerations are made as to how the IPE activity is arranged.

The different intersecting practices observed in the study all have their own shared repertoire [12]. Excerpt 1 show how the clinical practice calls for a negotiation that relates to how nurses usually divide patients between themselves during morning work. It also relates to how a ward round is expected to be conducted, and that a nurse should be the one to recount the current state of the patient´s condition. Roots run deep within healthcare, and traditions have an impact on interprofessional collaboration in various ways, such as hierarchies and cultural barriers [31, 32].

During the negotiations, students acted as brokers by connecting knowledge from different CoPs. Excerpt 2 exemplifies this. The negotiation between the medical and nursing students includes arguments derived from their respective profession-specific CoPs. Afterwards, the students expressed that they had to scrutinize their own arguments to see how valid they were. Other studies have also shown that students can turn into brokers during IPE [33]. This study confirms this by exemplifying how students’ brokering occurs during negotiations when collaborating in a clinical setting. Our findings add value to how students are able to discuss knowledge from different CoPs and relate them in a way that pushes their negotiations forward.

Being allowed time to negotiate proved to be a condition that enabled students in coming to terms with what should be done and by whom. When interrupted due to conflicting practices, students decided who should do what task based on availability rather than competence. Consequently, students’ chances to learn about roles and responsibilities are reduced. Other research studies [34] have also shown that having time to discuss patients with senior residents was perceived as positive for learning and being disrupted due to time schedules was perceived as negative.

The IPTW has proven to be a safe place for learning in previous research [35]. This study adds to this by showing how aligned practices can allow students to try out their arguments when negotiating with the others in a safe manner. However, the students did not always feel safe enough to shoulder responsibility for a task. This calls for efforts focused on how practices can align in a way that ensures safety and boosts students’ confidence.

Other efforts to ensure that students have time and feel safe enough to negotiate with each other should be directed to the supervisors. Overall, the role of the supervisor has shown to be important for students interprofessional learning in multiple studies [36–39] and there are lessons to be learned in how to effectively supervise students. However, many studies focus on concrete actions taken by the supervisors such as giving feedback, setting up a contract etcetera. Less focus has been paid to the supervisors’ role in ensuring that students have time and feels safe to negotiate. One aspect found in this study show that the clinical practice and the learning practice did not always align and hindered students’ negotiations. In a world where care and treatment times becomes shorter, students’ need for time to learn risks becoming compromised. To meet this challenge, research should not only focus on how supervisors support and hinder negotiations in the current situation. Research should also seek to find ways for supervisors to create a space that allows time even though there is not much of it, as well as increasing students’ sense of feeling safe.

The conflict between the interprofessional CoP and clinical or profession-specific CoPs also prompts a discussion on how to view IPE. Based on our findings, there are indications pointing at interprofessional collaboration being considered as the shared nursing care. While the profession-specific tasks are somewhat separate from the collaborative work, the two practices seemingly run in parallel rather than being aligned. However, how students understand the relationships between interprofessional collaboration and profession-specific tasks has not been the focus of our study and merits further investigation.

### Methodological considerations

Although the field of interprofessional research is reaching maturity [40] there is still a need for ethnographic research, studies that includes several contexts [40–42], and theory-based research [25, 40]. The argument being that the complexity of IPE calls for methods that address the full scope of an IPE activity. The holistic approach of ethnography, together with theory informing the analysis, is therefore considered to be well-suited for our aim of exploring the conditions for students to negotiate competences and responsibilities when working together as an interprofessional team.

The traditional way of conducting ethnography implies long periods of data collection to fully grasp the studied phenomena. This is a challenge for research on IPE. Many IPE activities are short in scope, usually varying between a day to a couple of weeks [43]. This is why focused ethnography serves research on IPE well [16]. Except for one data collection period that was interrupted due to the COVID-19 pandemic, the full length of each IPE activity was observed and therefore what the students go through. To ensure a rich dataset and not only a small case study, it was decided to do multiple rounds of data collection at the respective site. It was also decided, in line with Reeves’ ideas on future research [41, 42], to combine two sites as a way of enriching our understanding of the conditions under which students negotiate different tasks and competencies during interprofessional clinical placement.

The study was carried out in collaboration between two Nordic universities. Conducting team-based ethnography has challenged us but also enabled us to gain a better understanding of our aim. Pink argues that “the serendipity of anthropology happens from fieldwork to teamwork—the sharing and viewing of other researchers’ materials, discussion, checking things out with each other, and following through.” [21].

The challenge of our research design proved to be the asymmetrical data and can be considered a limitation of the study. Clerke and Hopwood [22] address asymmetry in team-based ethnography. They argue that asymmetry is not a reason to avoid team-based ethnography. On the contrary, asymmetry is ”valuable, creating enriched evidence and opportunities for analysis and representation.” (p. 39). However, the challenges lie in organising and managing the analysis to ensure trustworthiness. The asymmetry in this paper lies in terms of the varying duration of the IPE activity, how patients are included, and the different ways in which interviews/informal conversations were conducted. Naturally, our way of writing field notes also differs. Consequently, transferring aspects from one dataset to the other called for special efforts. Our team-based approach implied collegial support during the research process, and compensates by sharing responsibility for interpretations, analysis, and finalising the paper. The use of theory also helps to compensate for these limitations, as it enabled us to abstract and conceptualise the findings.

The Nordic collaboration entails working with data in a foreign language and from different cultural contexts. This implies potential barriers for the analysis, as there is a risk of misunderstandings and gaps in knowledge about the other language/cultural context. However, the two languages and cultures are similar in many ways. All authors are also experienced in understanding conversations in the other language and have come to understand nuances in language and cultural expressions. Whenever language and/or cultural barriers have occurred we have reached out to each other, cross-checking how to understand a situation.

Finally, Jensen et al. [44]. report that a limited amount of research papers include description of patient participation in the design and conduct of IPE studies. This goes for us as well to some extent. The COVID-19 pandemic hindered us from being able to observe students working together with patients during some of the data collection periods, and the negotiations that occurred in those situations were not observed. It is to be considered a limitation as the patient is a central part to both health care in general and interprofessional collaboration specifically, see IPEC [4]. However, the safety of everyone’s health had to be prioritised. We encourage research to focus more specifically on how the patient is involved when negotiating what to do and who should do it.

## Conclusion

In conclusion, students negotiate what should be done and by whom during interprofessional clinical placement. The findings show that this is a result of the intersection of different CoPs. When the CoPs aligned, they enabled students’ negotiations. When they were in conflict, they hindered or even interrupted negotiations. The paper show that this is a result of conditions concerning having time to negotiate and the feeling of being safe. Having time to negotiate enabled students to discern their respective competencies and conclude what to do and who should do it. When time was lacking, the negotiations were left stranded, resulting in students dividing tasks based on who was available rather than who was most competent. Feeling safe enabled the students to trust each other’s competencies and agree on what to do. When the students expressed feeling unsafe or not being competent enough, they tried to dodge tasks during negotiations and let someone else take responsibility.

The results imply that educators need to pay attention to how students relate to different CoPs as they negotiate what should be done and by whom. This concerns educators at all levels of the education system, from the faculty at the university who design IPE curricula to the supervisors of the specific IPE activity. Discussions on what CoPs should align and what CoPs can be allowed to be in conflict needs to be included in the evaluation on how best to develop clinical IPE.

## Data Availability

The datasets generated and/or analysed during the current study are not publicly available due to the ethical approval but are available from the corresponding author upon reasonable request.

## Abbreviations

CoP: Communities of practice
IPE: Interprofessional education
WHO: World Health Organization
